# Adverse events associated with administration of vasopressor medications through a peripheral intravenous catheter: do not confound access route and specific drug complications!

**DOI:** 10.1186/s13054-021-03605-6

**Published:** 2021-05-29

**Authors:** Romain Jouffroy, Benoît Vivien

**Affiliations:** 1 Service de Médecine intensive réanimation, Ambroise Paré Hospital, Assistance Publique – Hôpitaux de Paris, Paris, France; 2 Centre de recherche en Epidémiologie et Santé des Populations - U1018 INSERM, Paris Saclay University, Paris, France; 3 Institut de Recherche bioMédicale et d’Epidémiologie du Sport - EA7329, INSEP - Paris University, Paris, France; 4 SAMU de Paris, Service d’Anesthésie Réanimation, Hôpital Universitaire Necker - Enfants Malades, Assistance Publique - Hôpitaux de Paris, Université de Paris, Paris, France

To the Editor:

Owen et al. [1] recently reported in the Journal a low incidence of adverse events associated with peripheral intravenous vasopressor administration in a systematic review and meta-analysis.

While the authors must be congratulated for their interesting review on this relevant question, we think that their findings require some caution. First, none of the studies from the Owen et al. [1] report indicates the vasopressor concentration used, while it has been reported that peripheral intravenous vasopressor toxicity relies on vasopressor concentration, and that using lowered solutions of a lower concentration helps to improve tolerance [2], which noticeably increases the safety of peripheral intravenous vasopressor administration. Second, time to exposure directly affects incidence of complications: as stated by Owen et al. [1], among adult studies, the average duration of peripheral intravenous vasopressor administration is 12–24 h, which limits the risk. Third, the intrinsic peripheral venous toxicity is different among vasopressors, related to their respective potency [3]. Fourth, the authors only considered local anatomic events, but excluded potential systemic effects, such as tachyarrhythmia and hypotension. Nonetheless, these life-threatening complications may be due to incidents specifically related to the peripheral intravenous access, like for example, during arm compression for nursing. On the other hand, central venous access per se fully prevents occurrence of such mechanical events. Finally, it should be kept in mind that some complications are inherent to the access route itself: e.g., extravasation after peripheral intravenous catheter versus pneumothorax after sub-clavicular puncture. Conversely, local ischemia, necrosis, and tachyarrhythmia must be considered as complications specifically related to the vasopressor drug by itself, e.g., due to its intrinsic properties.

Beyond these limitations, we fully agree with Owen et al. [1] that the adverse events’ incidence and severity after peripheral vasopressors administration are low. Because the rate of adverse events resulting from peripheral vasopressor administration is not different from those from central lines [4], there is no reason to delay their use, especially for the sicker patients cared for in the prehospital and/or in-hospital setting [5].

## Data Availability

Not applicable.
